# Skin-to-skin contact after birth and the natural course of neurosteroid levels in healthy term newborns

**DOI:** 10.1038/jp.2016.268

**Published:** 2017-01-19

**Authors:** K R McCallie, N W Gaikwad, M E Castillo Cuadrado, M Aleman, J E Madigan, D K Stevenson, V K Bhutani

**Affiliations:** 1 Division of Neonatology, Stanford University, Palo Alto, CA, USA; 2 Department of Nutrition and Environmental Toxicology, University of California Davis, Davis, CA, USA; 3 Department of Veterinary Medicine, University of California Davis, Davis, CA, USA

## Abstract

**Objective:**

To determine the postnatal course of neurosteroid levels in relation to gender, mode of delivery and the extent of skin-to-skin (STS) contact during the first days of life in healthy term newborns.

**Study Design:**

Prospective observational study of 39 neonates in which parents recorded total duration of STS in the first 2 days and nine neurosteroids (dehydroepiandrosterone-sulfate, progesterone, pregnenolone, pregnenolone-sulfate, allopregnanolone, isopregnanolone, epipregnanolone, pregnanolone and pregnanolone-sulfate) were assayed from blood samples at birth and at 1–2 days of age.

**Results:**

All nine neurosteroid levels declined significantly during the first 2 days of life. Gender did not significantly affect the change in neurosteroid levels. The decline in neurosteroid levels was generally more pronounced in vaginal deliveries, and there was a trend toward a larger decline with more exposure to STS.

**Conclusion:**

Ongoing studies may better characterize the role of neurosteroids and the influence of STS in more critically ill and premature neonates.

## Introduction

Implementation of skin-to-skin (STS) contact, also known as Kangaroo Care, has been reported to benefit both term and preterm infants in short-term outcomes, such as breastfeeding establishment and blood sugar levels,^1^ as well as longer-term outcomes, such as infant growth, breastfeeding duration and mother–infant attachment.^2^ In particular, in preterm, very low birth weight infants, STS appears to improve neurodevelopmental outcomes, including accelerated neurophysiological brain maturation^3^ and improved emotional and cognitive regulatory capacity in infancy.^4^ However, there is a paucity of data on the biological mechanism of STS on neurodevelopmental outcome.

Neuroactive steroids, predominantly 5α-reduced pregnanes, cross the blood–brain barrier and affect brain development and functioning either by directly interacting with neurotransmitter receptors or through genetic mechanisms.^5, 6^ Neurosteroid biomarkers exert inhibitory and excitatory effects on neurotransmission to clinically impact stress, pain, cognition, memory and seizures; they can also produce neuroprotective and neurogenic effects.^7^ Neurosteroids exert these effects on the brain predominantly through GABA receptors but also via *N*-methyl-D-aspartate receptors, sigma-1 receptors, glycine receptors, microtubule assembly and myelin repair.^5, 6, 8^

Recent veterinary observations in birthing mammals have described neuroactive steroids as the cause of a neurological syndrome in newborn horses.^9^ In neonatal equine maladjustment syndrome (NEMS), behavioral abnormalities include altered states of consciousness, failure to bond with the mother, failure to nurse, wandering, lack of normal environmental responses and disorientation (see video at https://vimeo.com/190318093/06efdc9bfe).^10^ NEMS can be ameliorated using a ‘thoracic squeeze technique' (see video at http://vimeo.com/68410389),^11^ which is analogous to a ‘maternal embrace' in humans.

We hypothesized that STS contact after birth may affect the natural course of neurosteroid levels in human neonates. We studied nine of the neuroactive steroid compounds most commonly reported in the literature to act on the human brain.^5, 6, 7, 8^ These included inhibitory neurosteroids (allopregnanolone and pregnanolone), excitatory neurosteroids (pregnenolone-sulfate and dehydroepiandrosterone-sulfate (DHEAS)), neurosteroids with neuroprotective or neurogenic effects (pregnenolone and progesterone) and neurosteroids with mixed effects (isopregnanolone, epipregnanolone and pregnanolone-sulfate).^12^ Our objective was to determine the immediate postnatal time course of neurosteroid levels and study the change in neurosteroid levels in relation to infant gender, mode of delivery and the extent of STS during the first days of life in healthy term babies.

## Methods

This was a prospective observational study of healthy newborns in the delivery room and postpartum unit at Lucile Packard Children's Hospital Stanford during 2015 and 2016. Institutional Review Board approval was granted by Stanford University. Prospective participants were screened through the electronic medical record, and written informed consent was obtained from infants' parents prior to delivery, on admission or at preoperative appointments in the Labor and Delivery unit.

Inclusion criteria for study eligibility were inborn infants with gestational age ⩾37 weeks, birth weight ⩾2000 g, cardio-respiratory stability (as determined by clinician) and prior written informed consent. Exclusion criteria were congenital anomalies, cardio-respiratory instability, invasive ventilation, hypotension requiring pressor support, acidosis (pH<7.2), blood culture-positive sepsis, hypernatremia (serum sodium >150 mEq l^−1^) or other high-risk medical/surgical co-morbidities (as determined by the clinician).

For each infant, gestational age at birth in weeks, birth weight in grams, mode of delivery (vaginal or cesarean section) and infant gender were recorded. Parents were given logs to record the duration in minutes of all episodes of STS contact (defined as infant's bare chest touching the parent's bare chest, with infant either naked or in only a diaper) in the first 2 days of life. Parents were taught the definition of STS contact and instructed how to fill out the log by study personnel at the time of consent. The completed logs were then reviewed with families for any discrepancies or unclear entries at the time of collection by study personnel.

One milliliter of neonatal blood was collected in lithium heparin vacutainer and microtainer tubes (BD vacutainer, Franklin Lakes, NJ, USA) from the placental side of the cut umbilical cord at birth and from a routine heel stick concurrent with standard-of-care laboratory tests drawn between 24 and 48 h of age. Whole blood was centrifuged within 15 min of collection, and 300 μl of plasma was aliquoted, immediately frozen with liquid nitrogen and stored at −80 °C. Plasma samples were packaged with dry ice and shipped overnight to the Gaikwad Laboratory at the University of California Davis, Davis, CA, USA where samples were assayed for nine neuroactive steroids.

Plasma samples were extracted with little modification and subjected to ultra-performance liquid chromatography tandem mass spectrometry analysis as described previously^13^ using a Waters Acquity UPLC system connected with the high performance Xevo-TQ mass spectrometer. Resulting data was processed by using the TargetLynx 4.1 software (MassLynx Mass Spectrometry Software. 2007. Version 4.1; Waters, Milford, MA, USA), and all neurosteroid concentrations are reported in pg ml^−1^.

Owing to the large range and non-normal distribution of the steroid levels, statistical analyses were carried out with non-parametric tests at a significance level of *P*=0.05. The Wilcoxon signed-rank test was used to compare steroid levels at birth with those at 1–2 days of age. The two-sample Wilcoxon rank-sum (Mann–Whitney) test was used to compare the changes in steroid levels in the first 2 days based on infant gender, mode of delivery and duration of STS. Statistical analyses were performed using Stata 13 (Stata Statistical Software, 2013, Version 13, StataCorp LP, College Station, TX, USA).

## Results

We approached 90 expecting mothers and obtained 49 consents. Two infants were withdrawn owing to admission to the neonatal intensive care unit for cardio-respiratory instability. Forty-seven infants were enrolled in the study and 87 samples were assayed. We analyzed 39 infants with gestational age ⩾37 weeks (that is, full term) who had complete data, which included a cord blood sample from birth, a blood sample from 1–2 days of age and a completed STS log. Of the eight remaining infants who were excluded, one had gestational age <37 weeks, three were missing a cord blood sample and four were missing a blood sample from 1 to 2 days of age.

For the 39 infants, the mean birth weight was 3383 g (s.d. 507), and the mean gestational age was 38.8 weeks (s.d. 0.8). Twenty-four (62%) of the infants were male, and 30 (77%) were delivered by c-section. Twenty-eight (72%) of the deliveries were scheduled c-sections without preceding labor. Steroid levels after birth were collected concurrently with routine hospital blood draws, which were performed at a mean age of 26 h (range 24–45 h). Total duration of STS episodes in the first 2 days of life ranged from 0 to 1530 min with median 346 min (interquartile range (IQR) 450). For c-section versus vaginal deliveries, the median STS duration in the first 2 days was 462 (IQR 495) versus 240 (IQR 146) minutes (*P*=0.03, using the two-sample Wilcoxon rank-sum test).

**Figure 1:**
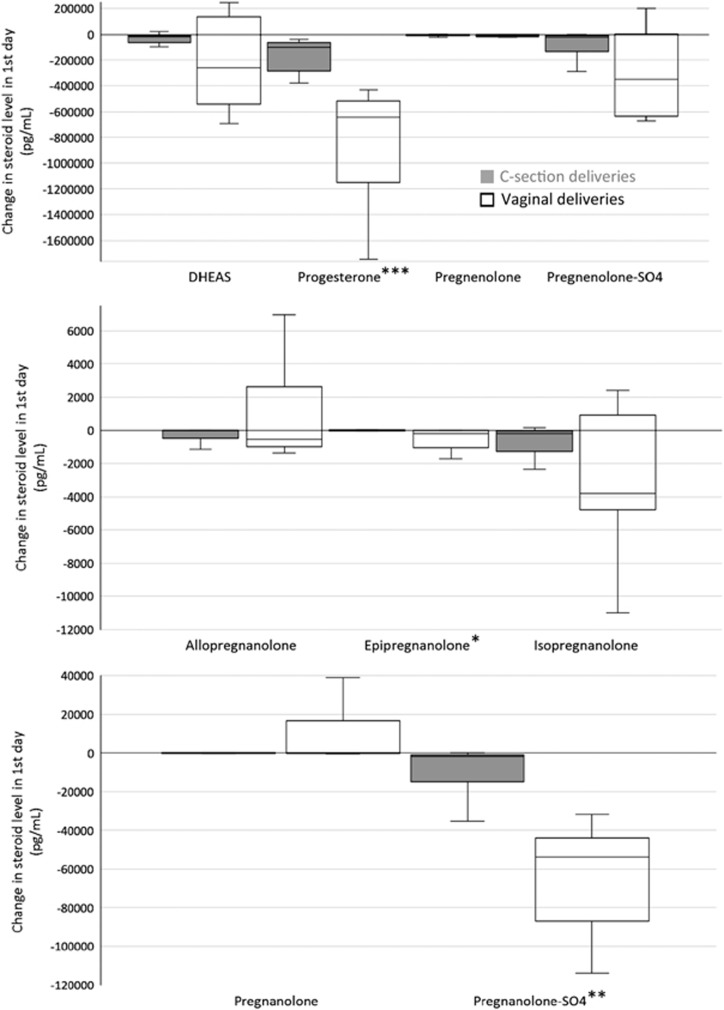
Change in neuroactive steroid levels over the first 2 days of life based on the mode of delivery. Boxplots show median, interquartile range, upper and lower adjacent values. Changes in steroid levels were compared using the two-sample Wilcoxon rank-sum test (*P<0.05, **P<0.001, ***P<0.0001). DHEAS, dehydroepiandrosterone-sulfate.

**Figure 2:**
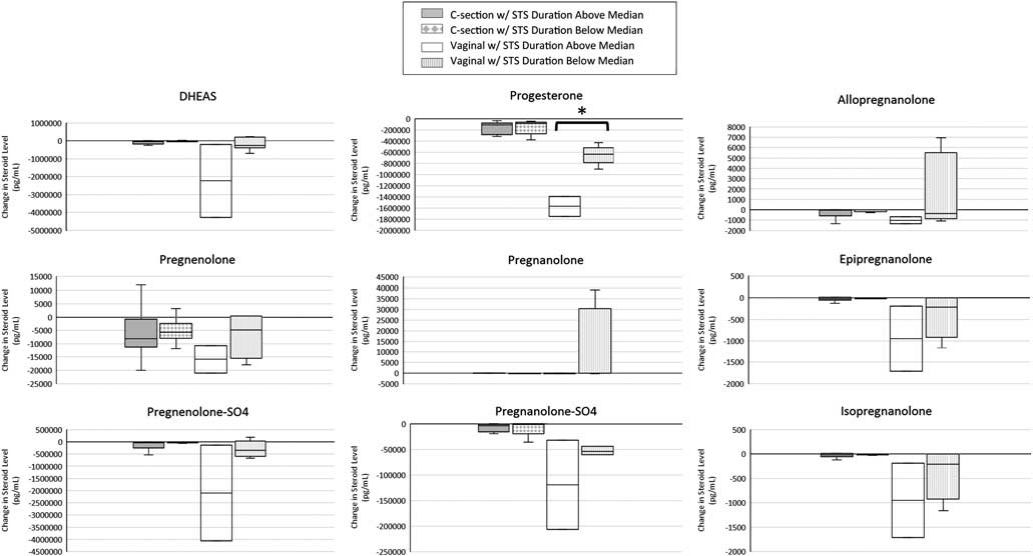
Effect of skin-to-skin (STS) duration on change in neuroactive steroid level in first 2 days of life based on the mode of delivery. Boxplots show median, interquartile range, upper and lower adjacent values. Median duration STS in the first 2 days of life=346 min. Changes in steroid levels were compared using the two-sample Wilcoxon rank-sum test (*P<0.05). DHEAS, dehydroepiandrosterone-sulfate.

Table 1 shows the changes in steroid level from birth to 2 days of age for the nine neurosteroids assayed. Changes in neurosteroid levels in the first 2 days were not affected by infant gender for any of the nine neurosteroids (data not presented). Figure 1 describes the changes in neurosteroid levels over the first 2 days of life based on the mode of delivery. Table 2 shows the effect of STS duration (above or below the median 346 min) on change in neurosteroid levels over the first 2 days of life, and Figure 2 displays the effect of STS duration on change in neurosteroid levels by the mode of delivery.

## Discussion

This is one of the first studies, to our knowledge, to document the natural course of multiple neuroactive steroids over the course of the first 2 days of life in healthy term newborns. Earlier studies on the use of mass spectrometry for the detection of newborn steroid levels in congenital adrenal hyperplasia testing have reported reference ranges for day 3 of life^14^ or days 9–40.^15^

In our study, there was a significant decline in steroid level from birth to 2 days of age for all nine of the neurosteroids assayed. The change in steroid level over the first 2 days was affected by delivery mode for the neurosteroids progesterone, epipregnanolone and pregnanolone-sulfate, in which the decline was significantly more in vaginal deliveries than in c-section deliveries. A trend was also seen toward a larger decline in neurosteroid level in vaginal deliveries for DHEAS and pregnenolone-sulfate. Duration of STS had a significant effect on the change in pregnanolone for all deliveries and in progesterone for vaginal deliveries, with a trend toward a larger decline in neurosteroid levels with more exposure to STS.

These results provide a potential link between STS and improved neurodevelopmental outcomes via effect on neurosteroid levels. For example, pregnanolone enhances activity at the GABA receptor, which has a multitude of inhibitory effects in the brain. Acute stress elevates the levels of inhibitory neurosteroids such as pregnanolone, and these neurosteroids are known to counteract many of the effects of stress.^16^ The larger decline in pregnanolone level in the first 2 days with more exposure to STS may reflect a less intense stress response or faster recovery from the stress of birth in these infants.

Our results are consistent with veterinary studies of newborn horse foals with NEMS—the neurological disorder manifested by abnormal behavior shortly after birth. Horses are prey animals, and normal foals rapidly stand and nurse within 2–3 h of birth and stay close to the mare for protection.^17^ Foals with NEMS do not perform these activities appropriately, despite the fact that the foals' mothers are normal in behavior and attempt to bond normally with the foals. Foals with NEMS often experience an alteration in the normal birth process, including rapid delivery, dystocia or c-section, and show persistent elevations of five different neurosteroids compared with age-matched normal foals.^9^ Experimental infusion of the neurosteroid allopregnanolone into normal foals also produces the same neurological symptoms transiently.^18^ Veterinarians have treated foals with NEMS by squeezing them^11^ to mimic birth canal pressure, which produces a rapid reversal of the clinical signs and a decrease in the elevated neurosteroid levels over time. In human neonates, we found that three of the neurosteroids that we assayed declined more in the first 2 days when born vaginally rather than by c-section, consistent with the findings in foals. And our hypothesis that STS during the first days of life, akin to the ‘squeeze technique' in foals, affects the natural course of human neurosteroid levels in that first 2 days was demonstrated for pregnanolone and progesterone, with a similar trend toward a larger decline with more exposure to STS in the other neurosteroids as well.

A limited number of neuroactive steroids have been studied in human neonates. For example, pregnenolone concentrations in healthy human neonates display a rapid and significant fall in the first days of life in both full-term and late preterm infants.^19^ After 12 h, significantly higher levels for pregnenolone were found in late preterm infants compared with full-term neonates, which was proposed to reflect a response to the stress of delivery in the premature infant. Our results also indicated that levels of pregnenolone, as well as the eight other neurosteroids, declined significantly during the first days of life. Future studies are planned to include late preterm (34–36 weeks' gestation) and more premature infants (28–33 weeks' gestation) to determine the changes in neurosteroid levels based on the degree of prematurity and its resulting clinical treatments, which may preclude STS provision. Recent literature^20^ has also indicated that peripheral concentrations of neurosteroids may not correlate with central nervous system concentrations. Thus future studies, first in neonatal animal models, and then potentially in human neonates, would ideally include neurosteroid levels in cerebrospinal fluid when it is already being obtained for other diagnostic or therapeutic purposes.

Given the predominance in our study of c-section deliveries not preceded by labor, we had the ability to compare newborns who experienced the ‘squeeze' of a vaginal delivery to those who did not. We saw that, similar to foals, neonates born by c-section had less of a decline in three neurosteroids (progesterone, epipregnanolone and pregnanolone-sulfate). Thus the effect of delivery mode on the decline in neurosteroid levels in the first 2 days may be due to the lack of labor rather than the mode of delivery *per se*. Further investigations into the reasons for c-section, the duration of labor in both vaginal and c-section deliveries preceded by labor and preexisting maternal and fetal morbidities will be necessary.

The large differential in c-section deliveries (*n*=30) versus vaginal deliveries (*n*=9) and overall small sample size are potential limitations. However, differential in sample size increases the chances of a type II (false negative) error but not a type I (false positive) error. The unequal group sizes would have therefore favored not finding a difference in the decline in neurosteroid level based on delivery mode. Instead, we did find significant differences for progesterone, epipregnanolone and pregnanolone-sulfate. Sample size calculations (*α*=0.05, *β*=0.2) indicate that increasing the total sample size to 80–100 infants (with equal number of vaginal and c-section deliveries) would give power to detect significant differences in the change in steroid level based on delivery mode for two additional neurosteroids (DHEAS and pregnenolone-sulfate).

Parental report of STS contact in the first 2 days may have been a contributing factor to the small number of neurosteroids that showed a significant relationship between duration of STS and decline in steroid levels. Despite training of parents on the STS logs at study consent and review of the logs with families after completion, both under-reporting and over-reporting of STS duration are possible. However, parents in a postpartum unit where the mothers and babies are cared for in the same room are the best positioned observers and recorders of STS that we currently have. In the future, as STS becomes standard of care, documentation by medical personnel of STS duration in the electronic medical record will become more standardized or, potentially, wearable devices that track proximity and body temperature may assist in more objectively tracking STS.

Although there was a trend toward a larger decline in neurosteroid levels over the first 2 days associated with more exposure to STS, this relationship was only significant for pregnanolone in all deliveries and for progesterone in vaginal deliveries. Given the findings in foals, we would have expected that exposure to STS would have a more significant impact on newborns born by c-section, where they potentially did not receive as much ‘squeeze' or ‘conditioning' during the birthing process. We did not, however, see this association in our small sample, which may be explained by the fact that these were healthy newborns. Healthy newborns may not be the correct analog to the neurologically abnormal foals with NEMS who respond to the ‘squeeze technique', and it may be that STS has a much larger effect on newborns where the normal prenatal, perinatal and postnatal processes are altered. This includes premature newborns, newborns with birth hypoxia resulting in neurological impairment and other critically ill newborns in the neonatal intensive care unit.

In addition to expanding the study population to more critically ill and more premature infants, future studies should also extend the study period beyond the first days of life. In particular, tracking the levels of neurosteroids along with the ‘dose' of STS contact in the first days to weeks to months of life could lead to the discovery of a biomarker for STS contact, which optimizes the neurodevelopment of these vulnerable patients.

## Conclusion

Neurosteroid levels decline during the first days of life in healthy term newborns. This decline is generally more pronounced in vaginal deliveries, and there is a trend toward a larger decline with more exposure to STS. Ongoing studies may better characterize the role of neurosteroids and the influence of STS in more critically ill and premature neonates.

## Figures and Tables

**Table 1:** Change in neuroactive steroid levels over the first 2 days of life

*Steroid*	*Mechanism of action in the brain*^5, 6, 8^	*Median (IQR) change in the first 2 days (pg ml*^*−1*^)	P*-value*
DHEAS	GABA receptor; Sigma-1 receptor	−21 700 (201 000)	0.0004
Progesterone	Myelin repair; Sigma-1 receptor	−236 000 (353 000)	<0.0001
Pregnenolone	Microtubule assembly	−7300 (9550)	<0.0001
Pregnenolone-SO_4_	NMDA receptor; GABA receptor; Sigma-1 receptor	−27 600 (281 000)	<0.0001
Allopregnanolone (3α,5α-TH-PROG)	GABA receptor	−45.4 (680)	<0.0001
Isopregnanolone (3β,5α-TH-PROG)	GABA receptor	−328 (3190)	<0.0001
Epipregnanolone (3β,5β-TH-PROG)	GABA receptor	−14.6 (125)	0.007
Pregnanolone (3α,5β-TH-PROG)	GABA receptor; glycine receptor	−13.3 (120)	0.001
Pregnanolone-SO_4_	NMDA receptor	−5120 (50 700)	<0.0001

Abbreviations: DHEAS, dehydroepiandrosterone-sulfate; IQR, interquartile range; NMDA, *N*-methyl-D-aspartate. Steroid levels from blood samples at birth and at 1–2 days of age were compared using the Wilcoxon signed-rank test.

**Table 2:** Effect of STS duration on change in the neuroactive steroid levels over the first 2 days of life

*Steroid*	*STS duration above median (*n=*19)*	*STS duration below median (*n=*20)*	P*-value*
DHEAS	−38 500 (248 000)	−14 600 (160 000)	0.13
Progesterone	−143 000 (211 000)	−273 000 (503 000)	0.43
Pregnenolone	−8580 (9180)	−5260 (7820)	0.33
Pregnenolone-SO_4_	−35 500 (279 000)	−20 400 (284 000)	0.15
Allopregnanolone (3α,5α-TH-PROG)	−48.3 (692)	−22.7 (455)	0.15
Isopregnanolone (3β,5α-TH-PROG)	−403 (3140)	−127 (2390)	0.22
Epipregnanolone (3β,5β-TH-PROG)	−18.8 (133)	−8.13 (116)	0.79
Pregnanolone (3α,5β-TH-PROG)	−31.7 (114)	−5.21 (63.1)	0.046
Pregnanolone-SO_4_	−5190 (18 800)	−19 000 (55 500)	0.63

Abbreviations: DHEAS, dehydroepiandrosterone-sulfate; STS, skin-to-skin. Data are median (interquartile range) change in the steroid level over the first 2 days in pg ml^−1^. Median duration STS in the first 2 days of life=346 min. Changes in the steroid levels were compared using the two-sample Wilcoxon rank-sum test.
